# The impact of new transportation modes on population distribution in Jing-Jin-Ji region of China

**DOI:** 10.1038/sdata.2017.204

**Published:** 2018-01-23

**Authors:** Lizhe Wang, Lajiao Chen

**Affiliations:** 1 China University of Geosciences, School of Computer Sciences, No. 388 Lumo Road, Hongshan District, Wuhan 430074, China; 2 Chinese Academy of Sciences, No. 9 Dengzhuang South Road, Haidian District, Beijing, Beijing 100094, China

**Keywords:** Environmental chemistry, Environmental social sciences

## Abstract

This paper conducts a novel study in China’s Jing-Jin-Ji region to investigate the determinants of population distribution and short-term migration based on a comprehensive dataset including traditional census data, earth observation data, and emerging Internet data. Our results show that due to the high level of urbanization in this region, natural conditions are no longer the strongest determinants of population distribution. New transportation modes, such as high-speed rail, have arisen as a significant determinant of population distribution and short-term migration, particularly in large cities. Socio-economic factors such as GDP, investment, urbanization level, and technology, which are traditionally assumed to govern population distribution and short-term migration, have less influence although education still remains an important factor affecting population distribution. These findings will contribute valuable information to regional planning decision-making in the Jing-Jin-Ji region.

## Introduction

Rapid population growth in the 20th century has led to urgent population problems^1–5^, and regional unbalance of population distribution, with some areas densely populated and others thinly populated, is also a severe population problem. To address such problems, it is critically important to find out what factors influence population distribution^6–9^. Understanding the determinants of spatiotemporal population distribution is fundamental to addressing the population problems associated with social, political, and environmental disruptions^7,10–12^.

Population determinants can be classified as ‘push’ or ‘pull’ factors, according to push-pull migration laws^13,14^. Push factors are negative characteristics that encourage migration out of an area whereas pull factors are positive characteristics that draw migrants from less favorable areas. For example, a pleasant climate and job opportunities could be pull factors for a region while an unpleasant climate and economic poverty could be push factors. In the early rural stages of human development, environmental factors such as climate were the major determinants of population distribution. By contrast, in today’s highly industrialized society, economic factors could be the dominant determinants^15^. Likewise, different regions may be affected by different determinants due to different local conditions of development and natural resources.

Previous studies on the population determinants of China have mainly used Census data. These kinds of data can describe long-term population distribution well, but fail to reveal short-term migration. However, with the development of global positioning systems and Internet technology, new emerging data make it possible to capture population flow on a short timescale^16,17^.

The Jing-Jin-Ji region contains the country’s capital (Beijing) and is also the largest urban region in Northern China, which includes the provinces of Beijing, Tianjin, and Hebei along the coast of the Bohai Sea. The population distribution of this region is overwhelmingly unbalanced on a county scale. This unequal spatial distribution of the region has remained stable historically (relatively low in the northwest and high in the southeast)^18^. Hence, the question arises: what are the intrinsic factors that determine the spatial distribution of population in the long term, and what are the major factors that control population flow on a short timescale?

We conducted a study in the Jing-Jin-Ji region to identify the determinants of population distribution based on a comprehensive dataset, including traditional census data, earth observation data, and new emerging Internet data. The results show that both long-term population distribution and short-term migration are significantly affected by new transportation modes, including high-speed rail (HSR) and air travel. HSR exacerbates population agglomeration in very large cities in this region and has less effect on smaller cities in Hebei. However, the influence of HSR on large cities is not proportionate to the size of the city.

## Methods

### Study area

The Jing-Jin-Ji region is located the Northern China, and includes the provinces of Beijing, Tianjin, and Hebei, with an area of 217.16 thousand km^2^. The region consists of 66 districts, 22 cities, and 196 counties with a total resident population of 110.69 million. The Jing-Jin-Ji region is in the North China Plain, which is low and flat with an elevation of less than 50 meters. The topography is low-lying from northwest to southeast. It has a warm temperate continental monsoon climate with distinct season change and a mean annual temperature of 10–12 °C. The mean annual precipitation is about 356 mm, and most of the precipitation occurs in July and August. The region contains both the capital and the northern metropolitan region of China. It is the biggest urbanized region in Northern China and includes the economic region surrounding Beijing, Tianjin, and Hebei, along the coast of the Bohai Sea.

### Population data

The population data used in this study include the population distribution which is based on long-term and short-term migrations.

The household population data is used to describe population distribution. We collected household population data for 2013 at county level from the Statistical Yearbooks of Beijing, Tianjin, and Hebei. We attributed the census data to county boundaries and constructed population distribution data (Data Citation 1).

The short-term migration refers to population flowing out of or into the target region. In contrast to household population, it is difficult to obtain this kind of data as it is hard to record the moving population on a short time scale. With the advent of the Internet however, mobile devices become an important source to characterize residents’ geographic behavior. The ‘Baidu Migration Big Dataset’ is such a source, and was obtained from the mobile app of the Baidu map or other apps using the location-based service (LBS) technique, a software-level service that uses location data to control features. The locations of people who took their mobile devices (such as a mobile phone or pad) between 369 cities were recorded every hour from February 7, 2015 to May 16, 2015. The locations of people were recorded in these 369 major cities. Nearly 121 million people’s locations have been recorded, which accounts for 8.8% of the total population of China (1,375 millions). This is the first real-time, dynamic, and visual dataset of population flow on a national scale. Admittedly, there are some drawbacks of the dataset. Firstly, the daily flow of people who did not use a mobile device or the mobile app are not recorded, which could be a large number. Secondly, the data only records locations eight hours a day. It is obvious that a certain amount of population flow is not captured.

In this study, an index of mobility frequency is defined to describe short-term migration. Mobility frequency is the total number of people that flow into and out of the target region per day. We calculate the index by the following steps. Firstly, we sum up the hourly population inflow to a daily inflow and sum up hourly outflow to a daily out flow. Secondly, we sum up the daily inflow and outflow data to produce a total daily flow. Finally, we sum up the total daily flow data for the whole recording period (for example, from February 7 to April 30) and average the value by dividing by the number of days.

### Potential determinants of population

According to demography theories, there are a wide variety of factors that determine the spatial distribution of population^19–21^. Those factors can be summarized into two groups: 1) physical factors, such as climate, topographical characteristics, availability of resources, and vegetation conditions; 2) socioeconomic factors such as economy, investment, education, and healthcare. The potential determinants of population for the Jing-Jin-Ji region selected in this study are summarized in Table 1.

The physical environment indicators selected in this study can be summarized into three categories: climate, topography, and land use and land cover. Climate determines the population distribution at large scale and over the long term.

The Temperature Humidity Index (THI) is used to represent climate suitability^22^. Such an index can better represent the impact of human settlement on the climate compared with other standards of measurement such as the wind speed index or the human comfort index. The concept of THI was originally proposed by Thom^23^ to quantify climate suitability^23^. The index is calculated according to the following equation:

(1)THI=(1.8T+32)−0.55×(1−RH)×(1.8T−26)

**Figure 1:**
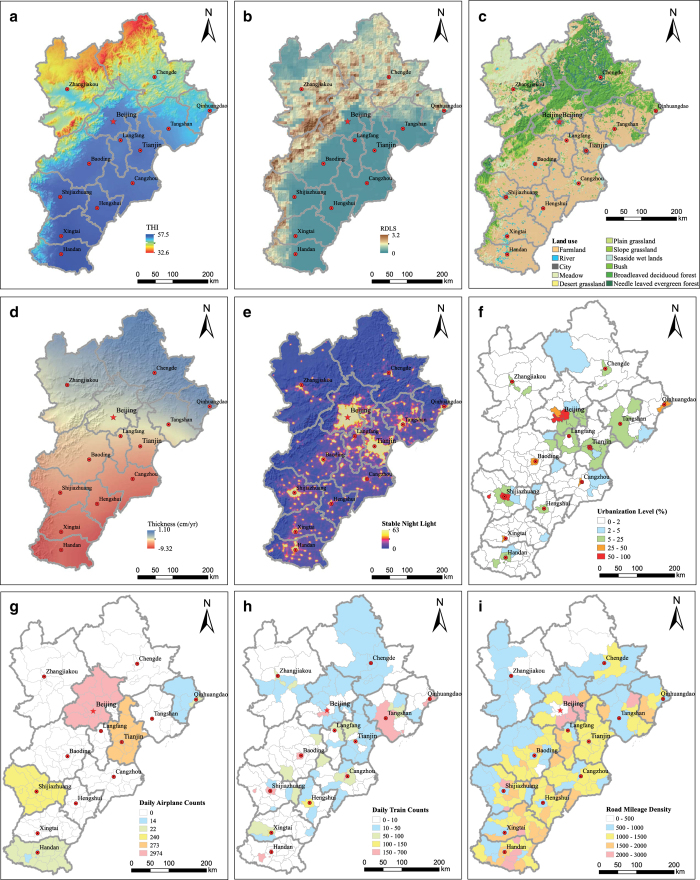
Indicators calculated from emerging data in Jing-Jin-Ji Region. (**a**) Spatial Distribution of THI in Jing-Jin-Ji Region. (**b**) Spatial Distribution of RDLS in Jing-Jin-Ji Region. (**c**) Spatial Distribution of Land Use and Land Cover in Jing-Jin-Ji Region. (**d**) Spatial distribution of TWS in Jing-Jin-Ji Region. (**e**) Spatial distribution of DMSP/OLS stable nighttime lights in Jing-Jin-Ji Region. (**f**) Spatial distribution of Urbanization Rate in Jing-Jin-Ji Region. (**g**) Number of daily airplanes in Jing-Jin-Ji Region. (**h**) Number of trains in Jing-Jin-Ji Region. (**i**) Number of high-speed trains in Jing-Jin-Ji Region.

Here, *T* is the average temperature and RH is the relative humidity. The spatial distribution of temperature and humidity were obtained from the Data Sharing Infrastructure of Earth System Science (http://www.geodata.cn/). The spatial distribution of *THI* is shown in Fig. 1a.

As people tend to live on gently sloping land, such as plains, topography plays an essential role at the large scale. To quantitatively represent the topographical condition, Relief Degree of Land Surface (RDLS) was selected to synthetically represent the altitude and incision depth of a region^22^. The RDLS is an effective means of describing the landform at a macro scale.

The RDLS is calculated according to the following equation^24^:

(2)RDLS=ALT/1000+((Max(H)−Min(H))×(1−P(A)/A))/500

The ALT is the average elevation of the neighborhood, and Max(H) and Min(H) are the maximum and minimum heights of the neighborhood, respectively. P(A) is the area of ground examined, and A is the area of the neighborhood, which was set to 25 km^2^. A 10 km^2^ window was established as the neighborhood extraction unit, from which Max(H) and Min(H) were extracted. Each window produced two data layers, and Min(H) was subtracted from Max(H) to obtain the height difference for each window.

These data were calculated in a previous study by our group^25^. Here, we used the relevant RDLS data for the Jing-Jin-Ji region (Fig. 1b).

The land use and cover data was acquired from the Global Land Cover 2000 Project (GLC 2000) (Fig. 1c), an international collaboration with the objective of providing a global land cover database for the year 2000 (ref. 26). These data were downloaded from the International Scientific & Technical Data mirror site at the Chinese Academy of Sciences.

With respect to natural resources, we mainly consider water resources in the region. Indicators of total amount of water resources and terrestrial water storage are used to characterize the water resources. Water storage is an important guarantee of population residence^27^. Terrestrial Water Storage (TWS) variation was calculated using data from the GRACE satellite mission, which was jointly sponsored by NASA and the German Aerospace Center and has been collecting data since mid-2002. Global-scale TWS maps have been derived using GRACE data (level 2, release 05) by three different institutes: the Center for Space Research (CSR), the Geo Forschungs Zentrum (GFZ), and the Jet Propulsion Laboratory (JPL)^28^. The three maps are all on a spatial resolution of 1°×1° from 2002 to 2013. In this study, we averaged the values from the three maps and interpolated the data over a 1 km^2^ grid (Fig. 1d).

The urbanization level is defined as the ratio of urban area to total area of a region. Urbanization level was calculated using the DMSP/OLS stable night-time lights database, which is run by the Air Force Space and Missile Systems Center (SMC) of the United States of America (http://ngdc.noaa.gov/eog/dmsp.html). We extracted the data of the Jing-Jin-Ji region from the global stable night-time lights dataset of 2013, which is shown in Fig. 1e. The intensity of night light is measured on a scale from 0 to 63, with a higher value indicating a higher level of human activity. Based on the existing studies, we set a threshold of 63 for Beijing and Tianjin with a high level of urbanization, and a threshold of 58 for Hebei with a low level of urbanization. Pixels that are equal to or higher than 63 are selected as urban pixels. Then we calculate the urban ratio at county scale. Urbanization level data is shown in Fig. 1f.

Number of daily airplanes is defined as the total number of airplanes arriving at and departing from a given airport each day. There are five airports in this region: Beijing Capital International Airport, Tianjin Binhai International Airport, ShiJiazhuang Zhengding International Airport, Qinhuangdao Airport, and Handan Airport. The number of daily airplanes is displayed in Fig. 1g.

The daily train density is defined as the number of trains of a given railway station. The data is acquired from the official website of the China Railway Service Centre (http://www.12306.cn). For each railway station in the Jing-Jin-Ji region, the number of each type of train was counted, including HSR, bullet rail, non-stop rail, express rail, and regular rail. Daily train density is displayed in Fig. 1g.

The road mileage data for each county in the Jing-Jin-Ji region were collected from the statistical information websites of Beijing, Tianjin, and Hebei. Road mileage densities were calculated by dividing total road mileage by the area of each county. The data are displayed in Fig. 1g.

Socioeconomic data, including health and medical conditions, education conditions, technology conditions, GDP per capita and investment were obtained from the statistical information websites of Beijing (http://www.bjstats.gov.cn), Tianjin (http://www.stats-tj.gov.cn), and Hebei (http://www.hetj.gov.cn).

### Data processing

Grids are used to extract information on population and its potential determinants in this study. Due to the fact that the data were originally collected from different sources with different formats and resolutions, we need to transfer the entire dataset into a single spatial resolution in raster format. Though it is preferable to conduct the data analysis at a fine spatial scale, too many pixels will bring huge computational cost. Therefore, we chose 10 km as the grid unit. Data processing is implemented by ArcGIS 10.2. GeoDetector software (http://www.geodetector.org/) is used to assess the contribution of potential determinants to population.

### Power of determinant

To discover the contribution of the potential population determinants and the interaction between them, we apply geographic detectors^29,30^. This approach is novel as it extracts the implicit interrelationships between risk factors and events without any assumptions or restrictions with respect to explanatory and response variables, which are difficult to model using classic epidemiological methods^31,32^. In this study, it is assumed that population will exhibit a spatial relationship to patterns of factors that contribute to the population dynamic. We overlay the population distribution on the geographical stratum of the potential determinant. The Power of Determinant (PD) method is used to assess the impact of population determinants on the spatial pattern of population^33^. A higher PD means that the risk factor has a stronger influence on the population distribution.

Here, we demonstrate how to quantify PD. Suppose the study area, Jing-Jin-Ji, has area A and population distribution *P*, with spatial units of $P_{i}\left(i=1,2,\ldots ,m\right)$. The potential determinants of population, or the environmental-socioeconomic factors (e.g., climate, resources, transportation), are overlaid on the population data. For a given environmental-socioeconomic factor, assume $D_{i}\left(i=1,2,\ldots ,L\right)$ are the attributions associated with this stratum. For a given attribution *D*_*i*_, certain grids of *P* will fall in the sub-region of *D*_*i*_. Then, the dispersion variance of *P* over sub-regions of attribute *D*_*i*_ is calculated, defined as $\sigma_{D,i}^{2}$. The PD to population distribution is given by

(3)PD=1−1nΣ2∑i=1LnD,iΣD,i2

where *n* is the total number of samples over the entire region for a given potential determinant, $n=\sum_{i=1}^{L}n_{D,i}$; L is the number of attributes for the given potential determinant; *nσ*^2^ denotes the variance of population of the entire area. The second term on the right hand side denotes the ratio of the *n*_*D,i*_ weighted divisional variations $\sigma_{D,i}^{2}$ to the global variance of population in the study area.

*PD*∈[0,1], where 1 indicates that the indicator completely controls the distribution of population while 0 means that the indicator has no impact on population distribution.

### Data availability

This dataset (Data Citation 1) includes spatial data (in ArcGIS shapefile format) of population distribution and determinants of population. The population data contain population distribution data at county level from 1991 to 2013 and short-term migration at city level. A table (in Excel format) associated with the attributes of the above data is also provided. The data of population determinants include the fowling ones: 1) physical factors, such as climate, topographical characteristic, availability of resources, land use, and land cover; 2) socioeconomic factors such as economy, education, and transportation. The physical factors are provided in the format of an ArcGIS grid while the socioeconomic factors are provided in ArcGIS shapefile format.

### Code availability

The GeoDetector software is used for data processing. This software is available at the website http://www.sssampling.org/Excel-geodetector.

## Results

### Spatial distribution of population and short-term migration in the Jing-Jin-Ji region

**Figure 2:**
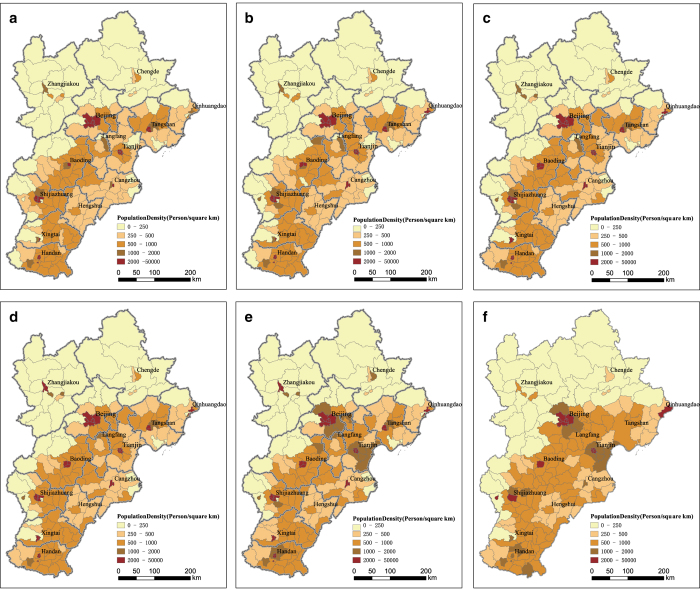
Spatial distribution of population in Jing-Jin-Ji Region. (**a**) Population density in 1991. (**b**) Population density in 1995. (**c**) Population density in 2000. (**d**) Population density in 2005. (**e**) Population density in 2010. (**f**) Population density in 2013.

Figure 2 shows the household population distribution of the Jing-Jin-Ji region from 1991 to 2013. Increasing from 81.03 million with a population density of 373 persons per square kilometer in 1991, household population reached 109.2 million with a population density of 502 persons per square kilometer by 2013. The regional population distribution is overwhelmingly unbalanced on the county scale. Population density varies from 46 to 36, 529 persons per square kilometer (Fig. 2). Population densities of Beijing, Tianjin, Shijiazhuang, and Baoding rank in the top three, exceeding 2000 persons per square kilometer. Population density stayed low in the northwest and high in the southeast from 1991 to 2013, which is consistent with the topographical features of the region.

### Short-term migration (population flow)

**Figure 3:**
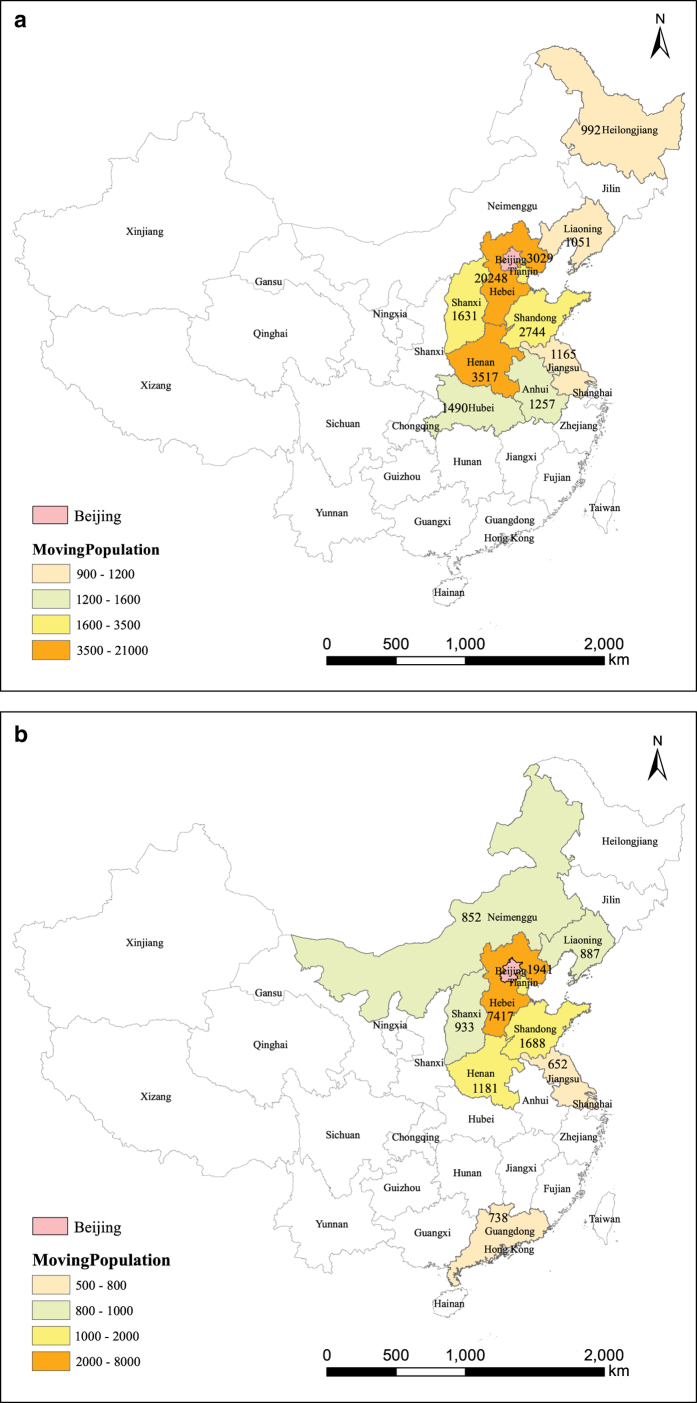
Top ten provinces with population moving of Beijing at 8:00 am on Feb.7th 2015. (**a**) Top ten provinces with population flowing in Beijing. (**b**) Top ten provinces with population flowing out Beijing.

Short-term migration refers to the population flowing out of or into a target region. In contrast to household population, it is difficult to obtain, due to the fact that it is hard to measure population movement rapidly or in real time. In early 2015, Baidu Inc. provided hourly migration data among provinces and cities, called the ‘Baidu Migration Big Dataset.’ Figure 3 illustrates population flow in and out of Beijing at 8:00 am on February 7th, 2015. The top 10 provinces from which population was flowing into Beijing (Fig. 3a) and the top 10 provinces into which population was flowing from Beijing (Fig. 3b) are both provided.

**Figure 4:**
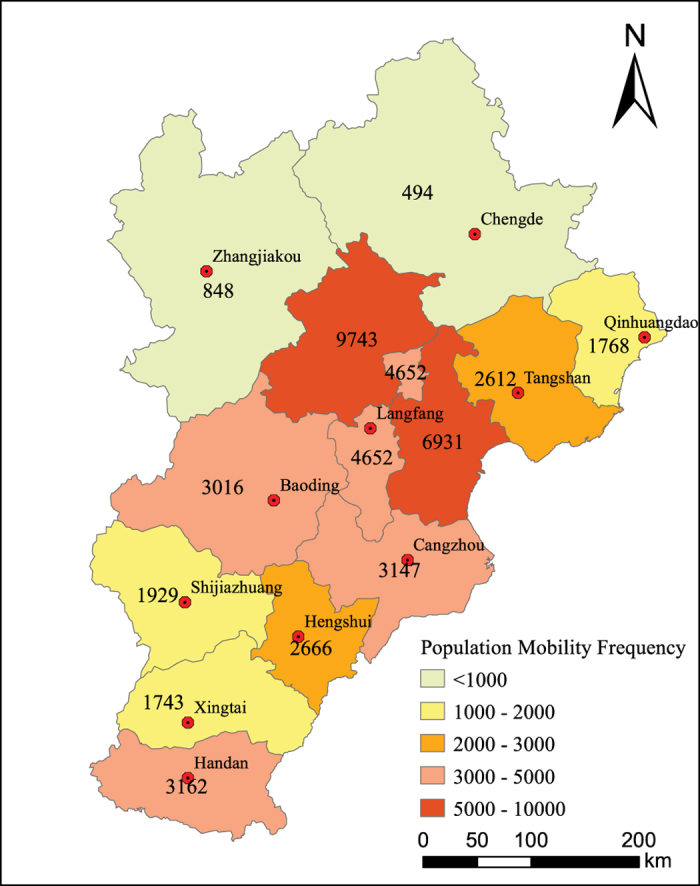
Population mobility frequency in Jing-Jin-Ji Region. Population mobility frequency refers to the average daily population flows in and out a given city. Dark color means high mobility frequency while light means low mobility frequency.

An index of mobility frequency, defined as the average population movement into or out of a given region in a given time period, was used to represent short-term migration in this study. The Baidu Migration Big Data from February 7 to April 30, 2015 was used to calculate mobility frequency in the Jing-Jin-Ji region. The estimated population mobility frequency of each capital city in the Jing-Jin-Ji region is shown in Fig. 4.

### Dominant population determinants

**Figure 5:**
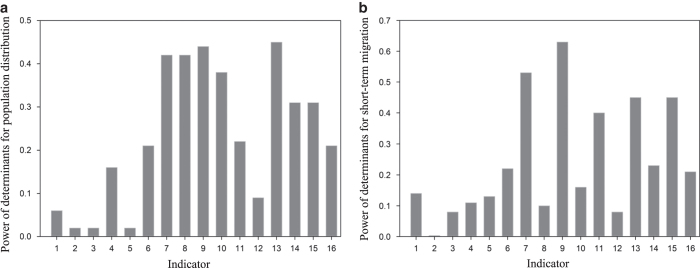
Power of determinants for population distribution and short-term migration. (**a**) Power of determinants for population distribution. The strongest determinants and their power values are the number of colleges (0.45), high-speed rail (0.44), number of daily airplanes (0.42), and number of trains (0.42). (**b**) Power of determinants for short-term migration. transportation determinants are dominant, including number of high-speed trains (0.63) and Number of daily airplanes (0.53).

Compared with socioeconomic factors, natural environment and resources have much less contribution to both population distribution and short-term migration. The availability of water resources has relatively high influence among natural determinants of population distribution; however, it has little influence on short-term population. This reveals that at the current stage of urbanization in the Jing-Jin-Ji region, natural conditions are no longer the dominant determinants of population distribution. The powers and rank of determinants of population distribution and short-term migration are listed in Table 2 and Fig. 5a,b.

For the distribution of population, the top four determinants are the number of colleges (0.45), HSR (0.44), number of daily airplanes (0.42), and number of trains (0.42). There are many universities in Beijing and Tianjin, which draw large populations; therefore, it makes sense that the number of colleges has a strong influence over resident population density. In addition, HSR, which has developed rapidly throughout China in recent years, appears to have had a significant influence on population distribution. A second cluster of moderately influential determinants includes technology and GDP indicators: number of patents accepted (0.31) and per-capita GDP (0.31). The third level of determinants includes urbanization and investment: total investment in fixed assets (0.21) and Urbanization level (0.21).

With respect to short-term migration, transportation indicators are dominant, including number of high-speed trains (0.63) and number of daily airplanes (0.53). These results reveal the significant impact of high-speed transportation modes on short-term migration. With the rapid development of transportation, travel time is shortened and accessibility is enhanced, thus people are more likely to travel or commute between cities. The second level of determinants includes number of colleges (0.45), per-capita GDP (0.45), and number of healthcare agencies (0.4). These results indicate that population flow among cites might be due in part to subsets of the population pursuing education, working, and seeking medical treatment. Minor determinants of short-term migration include technology, investment, and urbanization, as represented by the number of patents accepted (0.23), Urbanization level (0.22), and total investment in fixed assets (0.21).

Education clearly retains significant influence on population distribution, but traditional social and economic factors such as GDP, investment, urbanization, and technology, which have been assumed to dominantly control population distribution and population flow, have dropped in status. Transportation factors, especially high-speed transportation modes such as HSR, have become the most important determinants of resident population distribution and short-term migration.

### Multivariate interaction

The indicators that affect the distribution of population or short-term population flow might be independent, which means there is no interaction between a pair of indicators. The indicators also might complicated interact with each other. This means that one indicator could weaken or enhance the other indicator. To investigate whether two indicators are independent or not, we calculated the multivariate interaction between the indicators using the GeoDector. In this study, the impact of two indicators on population is defined as A and B, and the joint impact of the two indicators is defined as C=A∩B. Here, ∩ denotes the intersection between A and B. C=(A+B) denotes the two indicators are independent. C>(A+B) denotes the two indicators enhance each other in a non-linear way. If C<(A+B), there are three different scenarios: 1) C>(A and B) denotes the two indicators enhance each other; 2) C>(A or B), which is equal to C<(A or B), denotes one indicator is enhanced and the other one is weakened; 3) C<(A and B) denotes a non-linear weakening of the both indicators.

We investigated the joint impact of the socio-economic indicators on population distribution and short-term migration. We do not analysis the joint impact of natural environment and natural resources indicators here since they have lower power of determinant to population compared with socio-economic indicators. The results are listed in Tables 3 and 4. Tables 3 and 4 illustrate that all pairs have higher joint impact than the impact of individual indicator in forming the distribution of population and short-term migration. This means that all social-economic indicators enhance each other. Most of enhancements are found to be in a non-linear way with joint impact higher than the sum of individual impact. In addition, there are extremely strong enhancements between the indicators. For example, Number of regular primary schools and Total investment in fixed assets are found to promote the individual impact greatly in forming the population distribution (Number of regular primary schools ∩ Total investment in fixed assets=0.65>0.3=number of regular primary schools(0.09)+Total investment in fixed assets (0.21)). We also find that all of the joint impact of transportation indicator pairs on population distribution is lower than sum of individual impact, except for the pair of road mileage and number of daily airplane. This indicates that transportation indicator pairs enhance each other in a lower level than other pair of indicators.

### New transportation modes and their relationship to population

With the rapid development of transportation infrastructure, the means of transport in China have changed. HSR, a type of railway that operates significantly faster than traditional railways, is the most distinctive change in transportation in recent years. China began to plan for HSR in the early 1990s, and the Beijing-Tianjin intercity rail (Jing-Jin Rail) became the first HSR line to open to commercial operation in 2008. On December 25, 2012, the Beijing-Guangdong HSR, the world’s longest HSR line, went into operation from the country’s capital (Beijing) in the north to Shenzhen on the south coast. Since then, China has fully entered the HSR era. By the end of 2015, China had the world’s most extensive HSR network, with 19,000 kilometers of HSR lines, accounting for 60% of the world’s total at the time. Due to the advantages of HSR compared with traditional transportation modes, such as shortening travel time and increasing traffic accessibility, HSR significantly affects population flow. The Jing-Jin-Ji region is one of the areas with the highest HSR density in China (Table 5).

To further examine the impact of HSR on population distribution and regional population flow in the Jing-Jin-Ji region, we quantified population agglomeration and dispersion patterns before and after the existence of HSR lines. In this study, a population agglomeration index (PAI) is used as an indicator of spatial distribution patterns^34^. The PAI is defined as the ratio of the population fraction to the area fraction of a given city in a region, given by the equation:

(4)PAI=(PiPn)*100%(AiAn)*100%=Pi/AiPn/An

**Figure 6:**
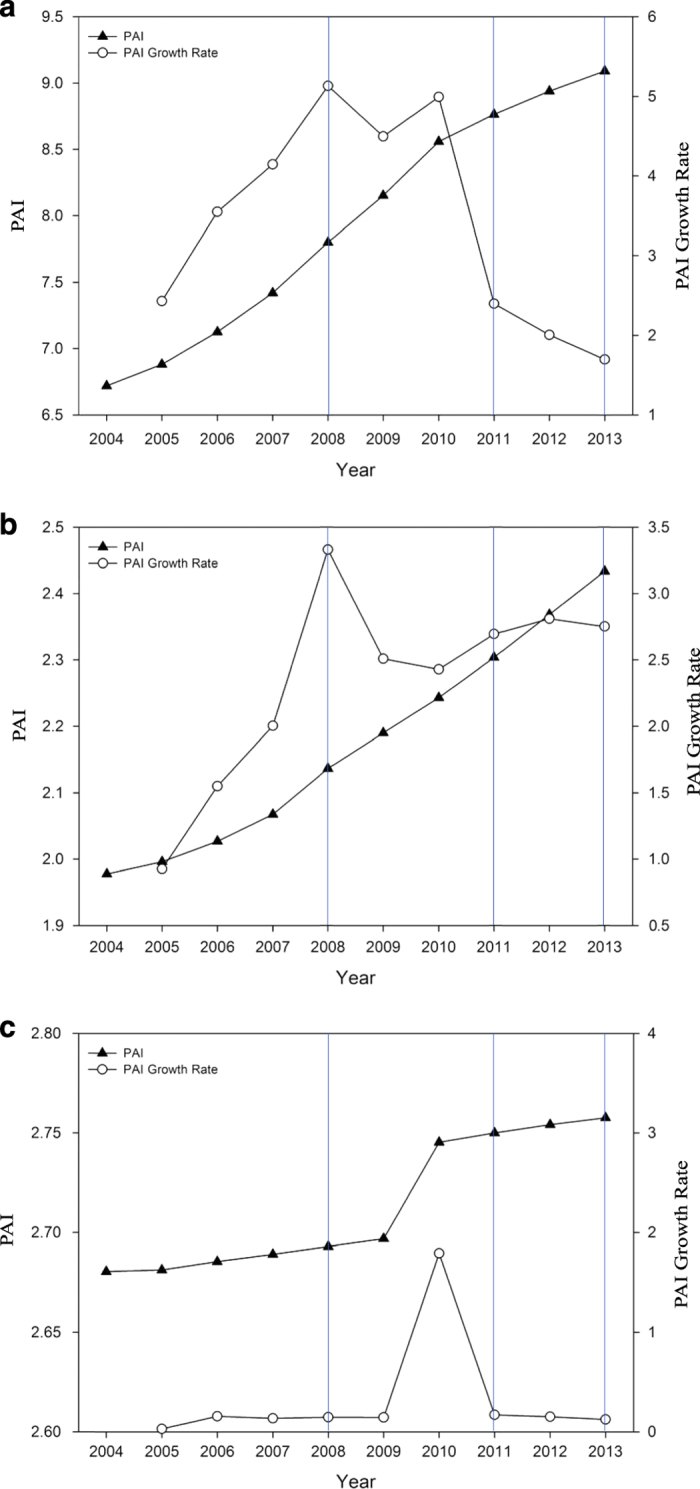
Population Agglomeration Index in the Jing-Jin-Ji Region. (**a**) Population Agglomeration Index of Beijing. (**b**) Population Agglomeration Index of Tianjin. (**c**) Population Agglomeration Index of Hebei.

Where *P*_*i*_ is the population of city *i*, *P*_*n*_ is the total population of the Jing-Jin-Ji region, *A*_*i*_ is the area of city *i*, and *A*_*n*_ is the total area of the area of the region. Such an index can quantify population agglomeration and dispersion of a certain city or county. According to previous studies, a PAI higher than 2 indicates population agglomeration in the given region, while a PAI lower than 0.5 indicates population dispersion in this region. PAI between 0.5 and 2 indicates an average population density. The PAIs of Beijing, Tianjin, and Hebei from 2004 to 2013 are shown in Fig. 6.

From Fig. 6 we can see that PAI increased rapidly in Beijing between 2004 and 2013. The PAI growth rate reached its first peak in 2008. As the Beijing-Tianjin intercity railway opened in 2008, this accelerated growth in population agglomeration can be ascribed to HSR. This provides further validation of HSR’s contribution to population flow to Beijing from other regions in China. In 2011, the year when the Beijing-Shanghai high-speed railway went into operation, the PAI growth rate was not as high as in 2008, suggesting that the Beijing-Shanghai HSR did not have the same impact on population agglomeration as the Beijing-Tianjin intercity HSR.

The fluctuation of PAI of Tianjin is strikingly similar to that of Beijing. It climbed rapidly from 2004 and reached its peak in 2008. It dropped in 2009 and then increased in the following year, reaching a peak in 2010. In contrast to Beijing, though, PAI growth rate decreased a little after 2008, but remained high from 2011 to 2013. It can be concluded that HSR has had a much greater and more lasting impact on population agglomeration in Tianjin than in Beijing.

**Figure 7:**
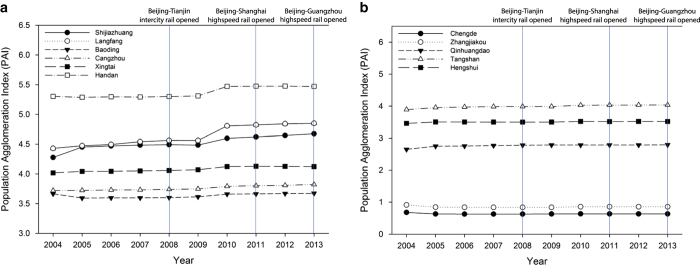
Population Agglomeration Index during 2004–2013. (**a**) Population Agglomeration Index of HSR-station cities during 2004–2013. (**b**) Population Agglomeration Index of non-HSR-station cities during 2004–2013.

The PAI of Hebei remained stable from 2004 to 2013, except for a slight increase in 2010. As the Beijing-Tianjin intercity railway only connects Beijing and Tianjin, population agglomeration has been limited in Hebei since 2008. To check whether the growth of PAI in 2010 was related to the construction of the Beijing-Shanghai and Beijing-Guangdong HSR lines, we further examined the PAI of the cities that had stations on these HSR lines. As shown in Fig. 7a,b, the PAI of cities with Beijing-Shanghai or Beijing-Guangdong HSR stations increased slightly from 2010. The PAI of those cities without high-speed rail had little or no change from 2004 to 2013, indicating that HSR has had a slight impact on population in Hebei, especially in cities with HSR stations.

The relationship between HSR and population in different cities in the Jing-Jin-Ji region reveals that HSR increased population accumulation in large cities in this region; however, HSR had little effect on small cities. Surprisingly, the influence on large cities was not proportional to the size of city. On the contrary, HSR affected Tianjin more strongly than Beijing, though the two cities are both important station cities for the Beijing-Tianjin and Beijing-Shanghai high-speed rail lines.

## Discussion

Identifying determinants of population distribution is essential for the study of demography and to better understand and respond to population problems. Here we present a novel study of population determinants in the Jing-Jin-Ji region based on a comprehensive dataset including traditional census data, earth observation data, and new emerging Internet data.

Our results reveal that due to increasing urbanization in the Jing-Jin-Ji region, natural conditions have little impact on population distribution. Though education remains an important influence on population distribution, traditional social and economic factors such as GDP, investment, urbanization, and technology, which are often assumed to be dominant determinants of population distribution and flow, have less influence compared with transportation. Meanwhile, new transportation modes such as HSR have arisen as the dominant determinant of both long-term population distribution and short-term migration. HSR exacerbates population agglomeration in the largest cities in this region but has less effect on smaller cities in Hebei. However, the influence of HSR on very large cities is not proportionate to the size of the city. On the contrary, HSR has a much greater and more lasting impact on population agglomeration in Tianjin than Beijing, though the two cities are both important station cities for the Beijing-Tianjin and Beijing-Shanghai HSR links. These findings will provide valuable information to regional planning decision-making.

## Additional information

**How to cite this article:** Wang, L. & Chen, L. The impact of new transportation modes on population distribution in Jing-Jin-Ji region of China. *Sci. Data* 5:170204 doi:10.1038/sdata.2017.204 (2018).

**Publisher’s note:** Springer Nature remains neutral with regard to jurisdictional claims in published maps and institutional affiliations.

## Figures and Tables

**Table 1:** Potential determinants of population in this study.

**Factors**	**Indicators**
*Natural environment and natural resources*	
Climate	Temperature Humidity Index (THI)
Topography	Relief Degree of Land Surface (RDLS)
Land surface	Land use and land cover
Water resources	Total amount of water resources
	Terrestrial Water Storage (TWS) variations
*Social-economic condition*	
Urbanization	Urbanization level
Transpiration	Number of daily airplanes
	Number of trains
	Road mileage
Health and medical condition	Number of health care agencies
Education	Number of regular primary schools
	Number of colleges
Technology	Number of three kinds of patent accepted
GDP	Per Capita GDP
Structure of industry	Ratio of third industry
Investment	Total investment in fixed Assets

**Table 2:** Power of determinants for population distribution and short-term migration.

**Factors**	**Indicators**	**Power of determinants for population distribution**	**Power of determinants for short-term migration**
*Natural environment and natural resources*			
Climate	(1) Temperature Humidity Index (THI)	0.06	0.14
Topography	(2) Relief Degree of Land Surface (RDLS)	0.02	0.003
Land surface	(3) Land use and land cover	0.02	0.08
Water resources	(4) Total amount of water resources	0.16	0.11
	(5) Terrestrial Water Storage (TWS) variations	0.02	0.13
*Social-economic condition*			
Urbanization	(6) Urbanization level	0.21	0.22
Transportation	(7) Number of daily airplanes	0.42	0.53
	(8) Number of trains	0.42	0.10
	(9) Number of high-speed trains	0.44	0.63
	(10) Road Mileage	0.38	0.16
Health and medical condition	(11) Number of health care agencies	0.22	0.40
Education	(12) Number of regular primary schools	0.09	0.08
	(13) Number of colleges	0.45	0.45
Technology	(14) Number of three kinds of patent accepted	0.31	0.23
GDP	(15) Per Capita GDP	0.31	0.45
Investment	(16) Total investment in fixed assets	0.21	0.21
Factors contains the natural environment and natural resources and social-economic condition two categories of 11 small classes. Indicators refers to 16 clusters. The last two columns are power of determinants for population distribution and short-term migration.			

**Table 3:** Joint impact of social-economic indicators on population distribution.

	**(6) Urbanization level**	**(7) Daily airplane schedule**	**(8) Number of trains**	**(9)Number of high-speed trains**	**(10) Road mileage**	**(11) Number of health care agencies**	**(12) Number of regular primary schools**	**(13) Number of colleges**	**(14) Number of three kinds of patent accepted**	**(15) Per Capita GDP**	**(16) Total investment in fixed assets**
(6) Urbanization level	—										
(7) Daily airplane schedule	0.71 (↑↗)	—									
(8) Number of trains	0.73 (↑↗)	*0.81* (↑↑)	—								
(9) Number of high-speed trains	0.71 (↑↗)	*0.68* (↑↑)	*0.77* (↑↑)	—							
(10) Road mileage	0.68 (↑↗)	0.81 (↑↗)	*0.68* (↑↑)	*0.80* (↑↑)	—						
(11) Number of health care agencies	0.53 (↑↗)	0.67 (↑↗)	0.73 (↑↗)	*0.63* (↑↑)	0.69 (↑↗)	—					
(12) Number of regular primary schools	0.60 (↑↗)	0.77 (↑↗)	0.69 (↑↗)	0.71 (↑↗)	0.76 (↑↗)	0.59 (↑↗)	—				
(13) Number of colleges	0.72 (↑↗)	*0.59* (↑↑)	*0.72* (↑↑)	0.74 (↑↑)	*0.78* (↑↑)	0.73 (↑↗)	0.78 (↑↗)	—			
(14) Number of three kinds of patent accepted	0.79 (↑↗)	0.73 (↑↗)	0.82 (↑↗)	0.79 (↑↗)	0.74 (↑↗)	0.69 (↑↗)	0.64 (↑↗)	0.82 (↑↗)			
(15) Per Capita GDP	0.58 (↑↗)	0.68 (↑↗)	0.70 (↑↗)	0.74 (↑↗)	0.68 (↑↗)	0.51 (↑↗)	0.58 (↑↗)	0.75 (↑↗)	0.72 (↑↗)		
(16) Total investment in fixed assets	0.59 (↑↗)	0.77 (↑↗)	0.75 (↑↗)	0.74 (↑↗)	0.75 (↑↗)	0.61 (↑↗)	0.65 (↑↗)	0.77 (↑↗)	0.64 (↑↗)	0.46 (↑↗)	
**Notes: ↑↑ denotes the two indicators enhance each other; ↑↗denotes a nonlinear enhancement of the two indicators.											

**Table 4:** Joint impact of social-economic indicators on short-term migration.

	**(6) Urbanization level**	**(7) Number of daily airplanes**	**(8) Number of trains**	**(9) Number of high-speed trains**	**(10) Road mileage**	**(11) Number of health care agencies**	**(12) Number of regular primary schools**	**(13) Number of colleges**	**(14) Number of three kinds of patent accepted**	**(15) Per Capita GDP**	**(16) Total investment in fixed assets**
(6) Urbanization level	—										
(7) Number of daily airplanes	0.78 (↑↗)	—									
(8) Number of trains	0.59 (↑↗)	0.72 (↑↗)	—								
(9) Number of high-speed trains	0.87 (↑↗)	0.87 (↑↑)	0.78 (↑↗)	—							
(10) Road mileage	0.79 (↑↗)	0.75 (↑↗)	0.47 (↑↗)	0.78 (↑↑)	—						
(11) Number of health care agencies	0.79 (↑↗)	0.78 (↑↑)	0.46 (↑↗)	0.79 (↑↑)	0.60 (↑↗)	—					
(12) Number of regular primary schools	0.83 (↑↗)	0.77 (↑↗)	0.47 (↑↗)	0.83 (↑↗)	0.65 (↑↗)	0.63 (↑↗)	—				
(13) Number of colleges	0.87 (↑↗)	0.66 (↑↑)	0.66 (↑↗)	0.87 (↑↑)	0.70 (↑↗)	0.70 (↑↑)	0.74 (↑↗)	—			
(14) Number of three kinds of patent accepted	0.86 (↑↗)	0.86 (↑↗)	0.61 (↑↗)	0.85 (↑↑)	0.62 (↑↗)	0.65 (↑↗)	0.56 (↑↗)	0.84 (↑↗)	—		
(15) Per Capita GDP	0.77 (↑↗)	0.78 (↑↗)	0.61 (↑↗)	0.77 (↑↑)	0.53 (↑↗)	0.50 (↑↑)	0.56 (↑↗)	0.82 (↑↗)	0.71 (↑↗)	—	
(16) Total investment in fixed assets	0.78 (↑↗)	0.77 (↑↗)	0.66 (↑↗)	0.78 (↑↑)	0.63 (↑↗)	0.52 (↑↗)	0.66 (↑↗)	0.74 (↑↗)	0.60 (↑↗)	0.46 (↑↗)	—
**Notes: ↑↑ denotes the two indicators enhance each other; ↑↗denotes a nonlinear enhancement of the two indicators.											

**Table 5:** List of the high-speed rails in Jing-Jin-Ji Region.

**High-speed rails**	**Operation time**	**Stations in JingJinJi Region**
Beijing-Tianjin intercity rail	August 1st, 2008	Beijing South, Yizhuang, Yongle, Wuqing, Tianjin
Beijing-Shanghai high-speed rail	June 30th, 2011	Beijing South, Tianjin South, Langfang, Cangzhou South
Beijing-Guangdong high-speed rail	December 26th, 2012	Beijing West, Zhuozhou East, Gaobeidian East, Baoding East, Dingzhou East, Shijiazhuang Airport, New Shijiazhuang, Gaoyi West, Xingtai East, Handan East
Beijing-Qinhuangdao high-speed rail	December 1st, 2013	Tianjin, Junliangcheng North, Binhai, Binhai North, Tangshan, Ruanhe, Beidaihe, Qinhuangdao
By the end of 2013, the Jing-Jin-Ji region included four HSR lines: the Beijing-Tianjin intercity rail, Beijing-Shanghai high-speed rail, Beijing-Guangzhou high-speed rail, and Tianjin-Qinhuangdao high-speed rail.		
